# Dataset for analysing the ESG-oriented technical efficiency of VNSI listed companies

**DOI:** 10.1016/j.dib.2023.109832

**Published:** 2023-11-26

**Authors:** Manh-Trung Phung, Van-Thi Dao, Khac-Thanh Mai

**Affiliations:** Financial Management Faculty-Vietnam Maritime University, 484 Lachtray, Haiphong, Vietnam

**Keywords:** Sustainability reports, Technical efficiency, ESG, Network data envelopment analysis

## Abstract

In tandem with the evolution of the “green economy and finance” paradigm, a number of sustainability assessment metrics tailored to businesses has been meticulously researched and refined. Among the established benchmarks, the Environmental, Social, and Governance (ESG) index has emerged prominently. With the strategic guidance of governments across diverse nations, this index has progressively assumed a central role as a guiding compass for enterprises with aspirations of sustainable development and the pursuit of long-term investment strategies coveted by discerning investors. The fundamental objective underpinning the dataset presented in this paper is to appraise the technical efficiency exhibited by listed companies on the Ho Chi Minh City Stock Exchange (HOSE), encompassed within the purview of the Vietnam Sustainability Index (VNSI). The assessment of information disclosure practices by these corporate entities adheres rigorously to the Global Reporting Initiative (GRI) standards of 2020. Complementing this are assorted financial metrics gleaned from financial and annual reports, underpinning an analytical framework predicated on Network Data Envelopment Analysis (NDEA), affording a ranking of corporate efficiency. This empirical dataset not only facilitates enhanced comprehension for investors and financial market overseers regarding the pivotal significance of ESG disclosures and practices but also serves as a catalyst for researchers, encouraging the exploration of broader research avenues or the development of novel, more efficacious evaluation frameworks.

Specifications Table

**Table:** 

Subject	Management and Decision sciences, Economics, Econometrics and Finance
Specific subject area	Decision Sciences, Climate and Environmental Finance, Economic Development and Growth.
Data format	Raw, Analyzed, and Filtered
Type of data	Table and Figure
Data collection	The data employed in this research can be classified into three primary categories: company financial data, sustainable development data, and marketabillity performance data. Financial data primarily derives from company financial reports. To acquire sustainable development data, particularly Environmental, Social, and Governance (ESG) metrics, we conducted targeted keyword searches within annual reports or dedicated sustainability reports. Information pertaining to the marketability performance under investigation was sourced from the Fireant metadata website.
Data source location	Financial data and ESG disclosure information are publicly available on the websites of the companies. Stock market data, reflecting the fluctuations of the companies' stocks, is stored on the Fireant website.
Data accessibility	Repository name: Mendeley Data Data identification number: 10.17632/s4n7397jvr.3 Direct URL to data: https://data.mendeley.com/datasets/s4n7397jvr/3 Instructions for accessing these data: access the direct URL and download the excel data file, namely ‘ESG-DEA DiB data outcomes en ver 3.xlsx’.

## 1 Value of the Data

• The assessment of sustainability reporting disclosure activities among enterprises has gained paramount significance within the financial market. This marks a pioneering initiative in Vietnam, wherein scores and the underlying methodology for appraising ESG disclosure scores of companies included in the VNSI index basket are publicly disclosed.
• This dataset assists financial market participants in comprehending the trajectory of ESG disclosure evaluation methodology advancement. From an investor's perspective, it serves as a valuable resource, enabling a more comprehensive assessment of a company and facilitating the development of sustainable, long-term investment strategies.
• The examination of business operational efficiency in the realm of ESG activities has emerged as an increasingly appealing subject of interest among scholars. Leveraging and advancing the dataset derived from this study, researchers can undertake the following objectives: (1) formulate theoretical models for assessing the efficiency of ESG disclosure and implementation practices within enterprises; and (2) extend empirical analyses to encompass the entire spectrum of publicly listed companies within the Vietnamese stock market.
• The data related to the evaluation of ESG information disclosure remains relatively limited, particularly in the context of developing countries. This data holds significant importance within the realm of data science. By referencing this dataset, the potential direction for future researches would relate to develop comprehensive ESG assessment models utilizing Big Data or Machine Learning algorithm.

## 2 Background

In the context of the increasingly fierce competition within the global economy, sustainable development has become an imperative requirement for businesses worldwide. Scholars have long been engaged in researching issues related to the disclosure and assessment of the impacts of Environmental, Social, and Governance (ESG) factors on the operational effectiveness of enterprises. For instance, Zahid et al. [1] comprehensively explored the facets of sustainability, including environmental, social, and governance dimensions, and examined the interplay of ESG with corporate financial performance (CFP) alongside auditing. The findings of this study provided a holistic overview by scrutinizing the influence of various ESG components, measures for enhancing financial performance, critical control variables, as well as the robustness of estimation techniques. Concurrently, Alexandra et al. [2] concentrated on investigating the effects of ESG factors on the operational efficiency of information technology companies (ITCs). Their research delved into the positioning of ITC firms in ESG rankings compared to other industries, thereby emphasizing key strengths and weaknesses within the ESG components.

Commencing on November 28, 1996, the State Securities Commission of Vietnam (SSC) was established by Government Decree No. 75/CP. Only two years later, on July 11, 1998, the Vietnamese stock market was officially inaugurated under Government Decree No. 48/CP. July 28, 2000, marked a significant milestone in the history of the Vietnamese stock market when the Ho Chi Minh City Stock Exchange (predecessor to the Ho Chi Minh City Stock Trading Center - HOSE) officially commenced operations with the first two traded stocks being REE and SAM. Building on this success, on March 8, 2005, the Hanoi Stock Trading Center (predecessor to the Hanoi Stock Exchange - HNX) was also officially introduced, becoming the listing center for small and medium-sized enterprises. With the miraculous development of the economy following the transition to a market-oriented mechanism, the Vietnamese stock market experienced rapid growth, attracting the attention of domestic and international investors. As of June 30, 2023, the market capitalization has reached 5.78 quadrillion VND, equivalent to 60.8% of GDP, a significant increase from the 1% observed in 2000. The scale of listing and trading registration in the market, as of the end of June 2023, reached nearly two quadrillion Vietnamese dong, encompassing 743 company stocks and ETFs listed on the two Stock Exchanges, and 866 stocks registered for trading on UPCoM (Unlisted Public Company Market).

## 3 Data Description

Evidently, the evaluation of operational efficiency in enterprises would exhibit significant deficiencies if not contextualized within a framework of sustainability analysis [3]. Within the Vietnamese landscape, the formulation of sustainable development indices stands as a pivotal undertaking, intimately linked with the innovation of financial instruments tailored for the “Green Capital Market.” This endeavor finds its roots in the Action Plan of the financial sector, designed to realize the National Green Growth Strategy by the year 2020. This strategic vision was set forth through Decision No. 2183/QD-BTC on October 20, 2015, promulgated by the Ministry of Finance. In July 2017, the Ho Chi Minh City Stock Exchange (HOSE) embarked on a noteworthy initiative following a period of collaborative research conducted in conjunction with the German International Cooperation Organization (GIZ) and the State Securities Commission (SSC). The result of this endeavor culminated in the establishment of the Vietnam Sustainability Index (VNSI). The index is calculated using the market capitalization-weighted method, adjusted for the free-float ratio, with a real-time calculation frequency of 5 s per iteration (Ho Chi Minh City Stock Exchange, 2017). Its constituents include the stocks of companies with the best sustainable development performance listed on HOSE within the VN100. For a company to gain admission to the exclusive VNSI index basket, its stock must undergo a rigorous screening process. This process entails the exclusion of enterprises deriving substantial revenues from sectors that are incongruent with the ideals of sustainable development. Such sectors include tobacco, nuclear energy, weapons, explosives, military equipment, gambling, casinos, alcoholic beverages (spirits), and other analogous activities. These exclusions are predicated on the premise that they run counter to the overarching objectives of sustainability. Subsequent to the exclusionary phase, HOSE employs a systematic methodology to compute and evaluate companies based on their sustainability development scores. This multifaceted assessment involves the administration of a sustainability development questionnaire, thoughtfully constructed to encompass the criteria delineated above. The ensuing evaluation process encompasses rigorous analysis, meticulous calculation, and the synthesis of scores for each dimension, denominated on a scale that extends to 100 percent. Twenty enterprises that achieve the highest sustainability development scores are deemed eligible for inclusion in the VNSI index basket. The top 15 stocks with the highest sustainable development scores will always be included in the VNSI basket. Stocks ranked 16th to 25th will be prioritized if they were already in the VNSI basket from the previous period, otherwise, new stocks will be considered until the basket reaches 20 stocks. In the event that a stock is removed from the VN100, it will also be excluded from the VNSI index on the effective date. In other words, the number of component stocks within this basket remains flexible, contingent upon the outcomes of the scoring mechanism and the prevailing conditions of the market. This index basket undergoes an annual review, conducted in the month of July (Table 1).

**Table 1:** List of companies in VNSI and their basic information (as of 2022/12/31).

No	Securities symbol	Company name	Outstanding Vol. (thousand)	Listing Vol. (thousand)	Chater capital (billiion)
1	AAA	An Phat Bioplastics Joint Stock Company	382,274	382,274	3822.0
2	BVH	BaoViet Holdings	742,323	742,323	7423.0
3	CTD	Coteccons Construction Joint Stock Company	74,414	78,831	788.0
4	DHG	DHG Pharmaceutical Joint Stock Company	130,746	130,746	1307.5
5	FPT	FPT Corporation	1269,969	1269,969	12,699.0
6	GEG	Gia Lai Electricity Joint Stock Company	341,249	341,249	3412.0
7	HDB	HCM City Development Joint Stock Commercial Bank	2892,551	2907,632	29,076.0
8	IMP	Imexpharm Pharmaceutical Joint Stock Company	66,705	66,705	667.1
9	MBB	Military Commercial Joint Stock Bank	5214,084	5214,084	52,140.8
10	NVL	No Va Land Investment Group Corporation	1950,104	1950,104	19,501.0
11	PAN	The PAN Group Joint Stock Company	208,895	216,295	2162.0
12	PLX	Vietnam National Petroleum Group: Petrolimex	1293,878	1293,878	12,938.8
13	PNJ	Phu Nhuan Jewelry Joint Stock Company	328,000	328,000	3281.0
14	SBT	Thanh Cong - Bien Hoa Joint Stock Company	740,501	740,501	7405.0
15	SSI	SSI Securities Corporation	1501,130	1501,130	15,011.3
16	STK	Century Synthetic Fiber Corporation Century Corp.	96,636	96,636	966.4
17	TPB	Tien Phong Commercial Joint Stock Bank	2201,635	2201,635	22,016.0
18	VIC	Vingroup Joint Stock Company	3813,936	3813,936	38,139.0
19	VNM	Viet Nam Dairy Products Joint Stock Company	2089,955	2089,955	20,899.0
20	VPB	VietNam Prosperity Joint Stock Commercial Bank	6713,204	6743,424	67,434.0

Despite its six-year implementation history, the dissemination of information regarding the VNSI index, coupled with investors' comprehension of the paramount importance of sustainable development, remains conspicuously deficient. A primary factor contributing to this conspicuous gap lies in the paucity of information available pertaining to the VNSI index. Remarkably, the updates found on the Ho Chi Minh City Stock Exchange (HOSE) website merely furnish a list comprising the 20 stocks included within this index basket. This glaring dearth raises legitimate queries concerning the transparency of the index grouping itself and the methodologies employed for the appraisal and ranking of sustainable development initiatives within the Vietnamese context. Therefore, the central aim of this research endeavor is to forge a methodological framework geared towards the assessment of ESG (Environmental, Social, and Governance) information disclosure by business enterprises. Subsequently, this framework endeavors to amalgamate variables encapsulating said information into a comprehensive model designed to evaluate the technical efficiency of these enterprises. This evaluation is underpinned by the Network Data Envelopment Analysis (NDEA) methodology. It is crucial to underscore that this dataset represents a pioneering initiative within the Vietnamese landscape, affording an unprecedented insight into the intricate landscape of non-financial disclosure activities encompassing ESG considerations. Additionally, it undertakes an appraisal of the efficacy of these initiatives through the discerning lens of the financial market.

Our dataset is encapsulated within an Excel file and stored in the Mendeley Repository (please refer to the Specifications table for the download link). This dataset encompasses seventeen sheets. The first sheet, namely the ‘GRI checklist’, illustrates the methodology for assessing ESG disclosure scores based on the GRI standards. The second sheet, ‘Descriptive’, comprises descriptive statistics data for all variables employed in the NDEA efficiency model. Subsequently, the following four sheets contain structured data according to the format of DEAfrontier software, utilizing both aggregate ESG scores and individual component scores (E, S, and G respectively). The subsequent four sheets present the result of efficiency scores and rankings (for the case of the aggregate ESG scores) under the assumptions of the 'Centralized' and 'Non-cooperative' network DEA model (explained later in the next section). The following six sheets demonstrate the results of efficiency assessments using the component scores (E, S, G) when either of the two stages is considered as the leader. The final data sheet consolidates the optimal multipliers corresponding to the variables employed in the efficiency model.

## 4 Experimental Design, Materials and Methods

The Global Reporting Initiative (GRI) standard is a globally recognized framework for sustainability reporting, that was introduced by the Global Sustainability Standards Board (GSSB) in 2016. It provides guidelines and principles for organizations to report on their economic, environmental, social, and governance (ESG) performance. It can be considered as the most effective international standard for preparing sustainable development reports.

This study, therefore, employs the GRI Standards Glossary (revised in 2020) to formulate a systematic approach for evaluating the sustainability reporting practices of enterprises listed on the Vietnam Sustainability Index (VNSI). The GRI Standards are structured into four distinct component groups, including (1) Governance approach; (2) Economic performance; (3) Environmental impact; and (4) Social relationship. In our pursuit of gauging the efficiency of Environmental, Social, and Governance (ESG) information disclosure, we deliberately narrow our focus to three specific information dimensions, namely governance, environmental, and social aspects. This endeavor is executed through the meticulous scrutiny of sustainability reports or annual reports, (if a sustainability report was unavailable), issued by the respective enterprises. For companies that do not publish dedicated sustainability reports, we employ a supplementary approach, entailing the identification of pertinent keywords within financial and economic news articles, primarily accessed through Vietnam's preeminent financial market information platform, Fireant. Each enterprise's adherence to the standards delineated in the GRI framework is evaluated by allotting a score of 1 in instances where information disclosure is detected, and a score of 0 in cases of non-disclosure. Employing this discerning methodology, we have derived comprehensive sustainability reporting scores, encompassing both overall disclosure practices and component-specific reporting scores (pertaining to governance, environment, and social facets) for each entity under scrutiny. These scores assume a pivotal role within the structure of our assessment model, serving as foundational indices for our evaluative framework.

Subsequent to quantifying the disclosure of ESG information, our study employs the Data Envelopment Analysis (DEA) method to scrutinize the efficiency of enterprises within the framework of ESG disclosure as an enduring objective. Among the array of efficiency analysis methodologies, DEA, which was originally introduced by Charnes et al. [4], emerges as one of the preeminent approaches. A distinctive feature of this method lies in its dispensation with the prerequisite of predefining a production function for the Decision-Making Units (DMUs) under evaluation. The conventional DEA model restricts its examination of the input and output parameters of a production process while regrettably bypassing the intricacies of internal processes. This model is mathematically formulated as a linear programming problem, delineated as follows:

(1) $$\begin{array}{ccc} & & \theta_{0}^{*}=Max\sum_{r=1}^{R}uy_{r0}\;\\ & & s.t.\;\;\;\;\sum_{i=1}^{I}v_{i}\;x_{i0}\;=1\\ & & \;\;\;\sum_{r=1}^{R}u_{r}\;y_{rj}\;\leq \sum_{i=1}^{I}v_{i}\;x_{ij}\;\;\;\;\;\;\;\;\;\forall j\\ & & \;\;\;u_{r}\;\;v_{i}\;\geq \varepsilon ; \end{array}$$

**Fig. 1:**
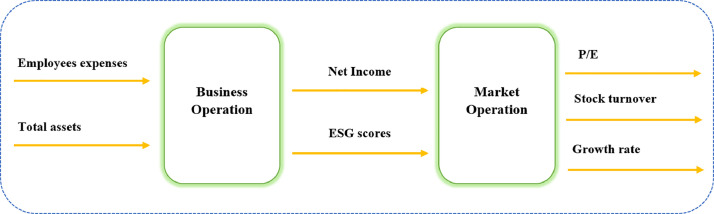
Technical efficiency evaluation network model under ESG-oriented.

In practice, the assessment of a DMU's efficiency extends beyond the mere consideration of input and output variables; it necessitates the inclusion of intermediate activities occurring within the operation. Fig. 1 illustrates a representation of a two-stage network evaluation model tailored, specifically, through the lens of sustainable development.

Given the diverse array of industries represented within the VNSI group, the assessment and comparison of the technical efficiency among these enterprises necessitates a deliberate selection of fundamental financial metrics. In particular, in our modeling framework, we have opted for two pivotal input variables: Labor Cost and Total Capital (aligned with the Cobb-Douglas production function model). It is noteworthy that these input variables not only serve as determinants of profitability, as indicated by the Net Income variable but also play a significant role in advancing sustainable development objectives, as embodied in the ESG variable. The final outcomes of this analytical process encompass the company's growth, gauged by the growth rate of total assets, and the appraisal of its market performance, a facet comprehensively assessed through two key variables: stock turnover and the P/E ratio. The descriptive statistics of all selected variables is illustrated in Table 2.

**Table 2:** Variables’ descriptive statistics.

Variables		Mean	Median	St.D.	Min	Max
Initial inputs	Labor Expense	3977.30	1118.00	5360.40	140.00	18,798.00
	Total Assets	174,198.56	50,066.50	236,114.06	2125.03	728,532.30
Intermediate measures	ESG	0.55	0.57	0.21	0.24	0.88
	E	0.45	0.42	0.24	0.09	0.91
	S	0.54	0.55	0.21	0.18	0.88
	G	0.69	0.76	0.24	0.10	0.97
	Net Income	6544.17	1988.65	11,331.88	34.83	43,681.30
Final outputs	P/E	25.80	20.69	19.15	5.48	71.76
	Turnoever	0.72	0.39	1.03	0.01	3.69
	Growth Rate	1.13	1.12	0.13	0.91	1.37

*Note: the units of labor expense, total assets, net income are billion VND, other variables are ratio*.

The challenge of evaluating such a model has been the subject of extensive scholarly inquiry [5]. Frequently, these investigations rely on certain assumptions regarding the interdependencies among distinct stages of the process (Phung et al. [6]; Zhang et al. [7]). One notable approach employed in such studies is the utilization of the “Noncooperative Network” model, which draws its foundations from Game Theory Principles. This particular model's fundamental tenet posits that within the aforementioned two stages, there exists a hierarchical dynamic: one stage assumes a “dominant” role as the leader, while the other stage assumes a complementary “follower” role. This conceptual framework, originally articulated by von Neumann et al. [8], has become a pivotal analytical tool in addressing efficiency concerns within network-based assessments.

Based on that premise, we can calculate the efficiency of the first stage, namely stage A as follows:

(2) $$\begin{array}{ccc} & & \theta_{0}^{A*}=Max\sum_{d=1}^{D}\eta_{d}\;z_{d0}\;\\ & & s.t.\;\;\;\;\;\sum_{d=1}^{D}\eta_{d}\;z_{d0}\;\leq \sum_{i=1}^{I}v_{i}\;x_{ij}\;\;\;\;\;\;\;\;\;\forall j\\ & & \;\;\;\sum_{i=1}^{I}v_{i}\;x_{i0}\;=1\\ & & \;\;\;\eta_{d}\;\;v_{i}\;\geq \varepsilon ; \end{array}$$

Subsequently, we can compute the efficiency of the second stage (stage B) based on the optimal value obtained from stage A, specifically as follows:

(3) $$\begin{array}{ccc} & & \theta_{0}^{B*}=Max\frac{\sum_{r=1}^{R}u_{r}\;y_{r0}\;}{\theta_{0}^{A*}}\\ & & s.t.\;\;\sum_{r=1}^{R}u_{r}\;y_{rj}\;\leq \sum_{d=1}^{D}\eta_{d}\;z_{d0}\;\;\;\;\;\;\;\;\;\forall j\\ & & \;\;\sum_{d=1}^{D}\eta_{d}\;z_{d0}\;\leq \sum_{i=1}^{I}v_{i}\;x_{ij}\;\;\;\;\;\;\;\;\;\forall j\\ & & \;\;\sum_{i=1}^{I}v_{i}\;x_{i0}\;=1\\ & & \;\;\theta_{0}^{A*}=\sum_{d=1}^{D}\eta_{d}\;z_{d0}\;\\ & & \;\;v_{i}\;\;\eta_{d}\;\;u_{r}\;\geq \varepsilon ; \end{array}$$

In a similar manner, we can assume that stage B takes on a dominant (leader) role. Therefore, the DEA efficiency of stage B will be calculated first, using the traditional CCR-DEA model. Then, the efficiency of stage A will be computed based on the constraints of the established efficiency model of stage B.

Table 3 presents the efficiency scores and rankings for the companies within the research sample. These efficiency scores are derived from distinct assessments of the relative importance of the two stages. Specifically, the first three columns depict the efficiency scores for Stage A (Business Operation), Stage B (Market Operation), and the overall efficiency under the assumption that Stage A holds greater significance (leader). Conversely, if Stage B is considered more pivotal, the corresponding efficiency scores are displayed in the subsequent three columns. The values enclosed in parentheses alongside the efficiency scores denote the respective ranking order of the companies. The variable representing the ESG disclosure score in this model corresponds to the average score across the three dimensions: E, S, and G. To economize on space, we have refrained from presenting the specific efficiency scores for these individual components here. Interested readers may consult the Excel data file for a more detailed analysis.

**Table 3:** Efficiency score and the corresponding ranking.

DMU	Stage A as Leader						Stage B as Leader					
	Stage A		Stage B		Overall		Stage A		Stage B		Overall	
*AAA*	0.17217	(15)	1.00000	(1)	0.17217	(8)	0.17217	(10)	1.00000	(1)	0.17217	(9)
*BVH*	0.04751	(19)	0.10936	(12)	0.00520	(15)	0.01657	(17)	0.37358	(16)	0.00619	(17)
*CTD*	0.63579	(7)	0.38850	(9)	0.24701	(6)	0.27490	(5)	1.00000	(1)	0.27490	(4)
*DHG*	1.00000	(1)	0.20748	(11)	0.20748	(7)	0.82920	(2)	0.29820	(17)	0.24726	(7)
*FPT*	0.67912	(6)	0.00517	(17)	0.00351	(16)	0.03013	(13)	0.29445	(18)	0.00887	(14)
*GEG*	0.37762	(8)	1.00000	(1)	0.37762	(3)	0.37762	(4)	1.00000	(1)	0.37762	(3)
*HDB*	0.14806	(16)	0.00330	(18)	0.00049	(17)	0.01743	(16)	0.39171	(14)	0.00683	(16)
*IMP*	0.76078	(5)	0.54880	(6)	0.41751	(2)	0.70098	(3)	0.70982	(9)	0.49757	(2)
*MBB*	0.20004	(13)	0.00108	(19)	0.00022	(20)	0.00556	(20)	0.79083	(8)	0.00439	(19)
*NVL*	0.20444	(12)	0.70357	(5)	0.14384	(9)	0.14279	(11)	1.00000	(1)	0.14279	(10)
*PAN*	0.26656	(10)	1.00000	(1)	0.26656	(4)	0.26656	(6)	1.00000	(1)	0.26656	(5)
*PLX*	0.12355	(17)	0.10529	(13)	0.01301	(14)	0.02681	(14)	0.26093	(19)	0.00700	(15)
*PNJ*	0.81131	(4)	0.02488	(15)	0.02019	(12)	0.11003	(12)	0.38793	(15)	0.04268	(12)
*SBT*	0.25248	(11)	1.00000	(1)	0.25248	(5)	0.25248	(7)	1.00000	(1)	0.25248	(6)
*SSI*	0.18781	(14)	0.53208	(8)	0.09993	(10)	0.17357	(9)	1.00000	(1)	0.17357	(8)
*STK*	1.00000	(1)	0.53873	(7)	0.53873	(1)	1.00000	(1)	0.53873	(11)	0.53873	(1)
*TPB*	0.04410	(20)	0.32153	(10)	0.01418	(13)	0.01926	(15)	0.54366	(10)	0.01047	(13)
*VIC*	0.06407	(18)	0.00570	(16)	0.00036	(18)	0.00592	(19)	0.46922	(13)	0.00278	(20)
*VNM*	1.00000	(1)	0.07781	(14)	0.07781	(11)	0.21400	(8)	0.24008	(20)	0.05138	(11)
*VPB*	0.31991	(9)	0.00073	(20)	0.00023	(19)	0.00918	(18)	0.48142	(12)	0.00442	(18)

For evaluating the degree of influence (relative importance) of variables within the operation chain, the optimal multipliers for the respective variables can be employed. Table 4 presents the average values of these multiplier coefficients, corresponding to the efficiency assessment models. It's also worth noting that these multipliers vary depending on the perceived importance of the stages in the network model.

**Table 4:** Variables's multipliers across DEA models.

Variables		Stage A as leader				Stage B as leader			
		E	S	G	ESG	E	S	G	ESG
Initial inputs	Labor Expense	1.2290	1.2014	1.3258	1.1967	11.2309	8.2713	6.7231	8.6143
	Total Assets	0.0513	0.0539	0.0490	0.0542	0.1619	0.1534	0.1267	0.1596
Intermediate measures	ESG	0.2217	0.2712	0.2266	0.2338	1.8815	1.6258	1.0171	1.5633
	Net Income	0.2746	0.2494	0.2431	0.2636	0.4249	0.3805	0.3906	0.3987
Final outputs	P/E	0.0003	0.0005	0.0005	0.0005	0.0028	0.0040	0.0029	0.0029
	Turnoever	0.0135	0.0120	0.0076	0.0125	0.0432	0.0760	0.0357	0.0607
	Growth Rate	0.0930	0.0909	0.0946	0.0826	0.3731	0.2999	0.2709	0.3002

## Limitations

The assessment of ESG disclosure scores for companies was conducted through keyword and boolean search methods. Specifically, we performed targeted keyword searches aligned with the criteria specified in the GRI Standards, amounting to over a hundred criteria in total. Given resource limitations, our data collection was confined to 20 publicly listed companies on the Ho Chi Minh City Stock Exchange, all of which are constituents of the VNSI index basket. To achieve an expanded database covering a wider range of listed companies across various stock exchanges in Vietnam, the development of advanced algorithms, including Big Data Analysis or Machine Learning, will be imperative for future research endeavors.

Furthermore, it is essential to emphasize that the outcome of the Data Envelopment Analysis approach provides relative efficiency assessments among Decision-Making Units (DMUs), primarily aimed at ranking purposes. In simpler terms, businesses are evaluated in comparison to the “best-practice performing” within the evaluated group of companies rather than being assessed for absolute efficiency.

## Ethics Statement

The authors have read and follow the ethical requirements for publication in Data in Brief and confirming that the current work does not involve human subjects, animal experiments, or any data collected from social media platforms. The authors have full permission to use the primary data.

## CRediT authorship contribution statement

**Manh-Trung Phung:** Conceptualization, Methodology, Software, Data curation, Writing – original draft. **Van-Thi Dao:** Data curation, Writing – original draft. **Khac-Thanh Mai:** Data curation, Supervision, Writing – review & editing.

## Data Availability

Dataset for analysing the ESG-oriented technical efficiency of VNSI listed companies (Original data) (Mendeley Data)
